# Effects of Er:YAG laser irradiation of different titanium surfaces on osteoblast response

**DOI:** 10.1007/s10856-021-06493-y

**Published:** 2021-03-06

**Authors:** Christian Wehner, Markus Laky, Hassan Ali Shokoohi-Tabrizi, Christian Behm, Andreas Moritz, Xiaohui Rausch-Fan, Oleh Andrukhov

**Affiliations:** 1 Division of Conservative Dentistry and Periodontology, University Clinic of Dentistry, Medical University of Vienna, Vienna, Austria; 2 Division of Orthodontics, University Clinic of Dentistry, Medical University of Vienna, Vienna, Austria

**Keywords:** Erbium laser, Osteoblasts, Titanium surface, Surface decontamination, Osteogenesis

## Abstract

The aim of this in vitro study was to evaluate the effects of erbium*-*doped yttrium aluminum garnet (Er:YAG) laser irradiation on titanium surface topography and the proliferation and differentiation of osteoblasts using standard clinical treatment settings. Er:YAG laser irradiation at two levels ((1): 160 mJ, pulse at 20 Hz; (2): 80 mJ, pulse at 20 Hz) was applied to moderately rough and smooth titanium disks before MG-63 osteoblast-like cells were cultured on these surfaces. Titanium surface and cell morphology were observed by scanning electron microscopy. Cell proliferation/viability was measured by CCK-8 test. Gene expression of alkaline phosphatase (ALP), osteocalcin (OC), osteoprotegerin (OPG), receptor activator of nuclear factor kappa-B ligand (RANKL), and collagen type 1 was measured by qPCR, and OPG and OC protein production was determined by enzyme-linked immunosorbent assay. Treatment with Er:YAG laser at 160 mJ/20 Hz markedly caused heat-induced fusion of titanium and cell condensation on moderately rough surfaces, but not in smooth surfaces. MG-63 proliferation/viability decreased after 5 days in moderately rough surfaces. The expression of ALP, OC, OPG, and collagen type 1 was unaffected by laser treatment at 160 mJ/20. Laser irradiation at 80 mJ/20 Hz enhanced RANKL gene expression after 5 days in moderately rough surfaces. Study results suggest that Er:YAG laser irradiation at clinically relevant setting has no essential effect on osteogenic gene and protein expression of osteoblasts. However, surface structure, cell attachment, and proliferation are influenced by both treatment protocols, which implies that caution should be taken in the clinical treatment of peri-implant diseases when Er:YAG laser is used.

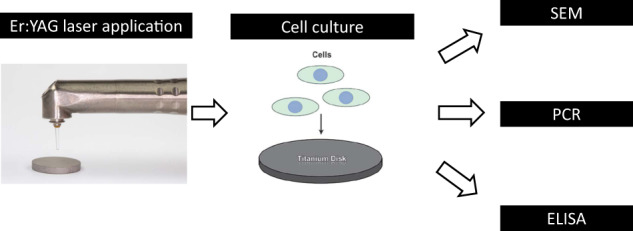

## Introduction

Endosseous implants nowadays represent a standard treatment procedure in clinical practice, demonstrating high long-term success rates [1, 2]. Titanium has shown long-term evidence as a safe material for dental implants and still is considered gold standard. In terms of tissue biocompatibility, surface topography is considered as a critical factor influencing the osseointegration process following implant insertion [3]. Preclinical studies have demonstrated that titanium implants with moderately rough surfaces exhibit both higher bone-to-implant contact and removal torque compared to smooth surface implants [4]. Profile roughness average (Sa) of 1–2 µm is suggested to provide optimal conditions for osseointegration, and the majority of commercially available implants nowadays match this roughness level [5]. In vitro, moderately rough surfaces promote osteoblast migration and the expression of osteogenic differentiation markers, whereas these functional parameters might be inhibited by extremely rough surfaces [6]. Despite positive effects on osseointegration, moderately rough surfaces also promote bacterial adherence and facilitate plaque accumulation, which is considered the primary etiologic agent for peri-implant diseases [7, 8].

Peri-implantitis is a site-specific inflammatory disease affecting peri-implant soft and hard tissues and might lead to implant loss. With a subject-based weighted mean prevalence of 19.83%, peri-implantitis represents a growing problem in dentistry, as numbers of dental implants placed are increasing [9]. Accumulation of plaque and the formation of biofilm on the implant surface promote the development of mucositis, which can progress to peri-implantitis without proper treatment [10]. Peri-implantitis treatment strategies, either surgical or nonsurgical, aim primarily to achieve sufficient surface decontamination [11, 12]. Although literature describes several approaches to treat peri-implantitis, there is no gold standard method defined clinically, as the treatment success often remains unpredictable [13–16].

Application of effects of erbium*-*doped yttrium aluminum garnet (Er:YAG) laser represents a promising tool for surface decontamination, which is a prerequisite in the treatment of peri-implant diseases [17]. This laser can be used for both nonsurgical and surgical approaches [18] and demonstrates superior effects with regard to bactericidal efficacy compared to conventional mechanical debridement using curettes [19]. Such decontamination strategy is especially useful on rough surfaces and/or between contaminated implant threads. An essential requirement for laser treatment protocols is the minimal collateral damaging effect on the implant surface, which could impair the re-osseointegration process.

To date, evidence on the impact of Er:YAG laser treatment of titanium surfaces on osseointegration generally and osteoblast response, in particular, is scarce. On the one hand, Er:YAG laser irradiation was shown to cause less temperature increases and profound alterations of titanium surfaces compared to the neodymium-doped: yttrium, aluminum, and garnet or CO_2_ lasers [20]. Few studies investigating the effect of Er:YAG laser irradiation suggest beneficial effects on osteoblast adhesion and proliferation [21, 22]. On the other hand, Er:YAG laser irradiation might cause adverse events, such as distinct alterations of titanium surfaces, when applied at high energy settings [23]. Findings by Galli et al. [24] suggest that Er:YAG laser irradiation of machined as well as on sandblasted and acid-etched titanium disks at 150 mJ/10 Hz pulse decreases proliferation of osteoblast-like Saos-2 cells. The same study further shows that titanium surface irradiation with Er:YAG laser at higher settings impairs the production of osteoprotegerin (OPG) [24]. Recent studies suggest that Er:YAG laser irradiation is also capable of debriding contaminated implant surfaces even at a low-energy level of 50, 60, or 120 mJ/pulse [25–27]. However, the effect of clinically relevant low-energy Er:YAG laser settings on osteoblast response, representing a crucial element for implant re-osseointegration, has not been investigated to date.

Therefore, the present study aimed to evaluate how the treatment of smooth and moderately rough titanium surfaces with Er:YAG laser at clinically relevant irradiation settings influences the response of osteoblast-like MG-63 cells cultivated on these surfaces.

## Materials and methods

Test titanium disks of 1 mm thickness and 15 mm in diameter were prepared from grade 2 unalloyed commercially pure titanium sheets. Pickled (PT) disks were degreased by washing in acetone and processed through a 2% ammonium fluoride, 2% hydrofluoric acid, 10% nitric acid solution at 55 °C for 30 s. The coarse-grit acid-etched surfaces (SLA) were prepared, as described previously [28, 29].

### Sample preparation and Er:YAG laser treatment

The laser used for experiments was a solid state Er:YAG (Fotona; 2940 nm, Ljubljana, Slovenia) with a wavelength of 2940 nm and a flexible arm. The titanium samples of both PT and SLA surfaces were irradiated at a distance of 2 mm with a conical fiber (Fotona Conical 8. 1,3–0,8, Ljubljana, Slovenia) held at 90 degrees, with a feed speed set at 1 mm per second and continuous water cooling. The application was moved in a standardized meander pattern to cover the entire surface, using a programmable mechanical device. Irradiation parameters were as follows:

- Group I (PT I, SLA I): 160 mJ, pulse at 20 Hz.
- Group II (PT II, SLA II): 80 mJ, pulse at 20 Hz.

Untreated PT and SLA surfaces served as controls.

Following laser treatment, each side of the titanium disks was exposed to UV light for 20 min for sterilization before cell cultivation.

### Cell culture

Osteoblast-like MG-63 cells (American Type Culture Collection, Rockville, USA) were used in the present study. Cells were cultured in modified Eagle’s minimum essential medium (MEM, Gibco, Carlsbad, USA) supplemented with 10% fetal bovine serum, penicillin (100 U/ml), and streptomycin (50 µg/ml) at 37 °C in a humidified atmosphere containing 5% CO_2_. Cells between the third and sixth passages were used in the experiments. Since MG-63 cells are an osteosarcoma cell line, no ethical approval was required.

### Cell proliferation/viability

Cell proliferation/viability was measured using a cell counting kit (CCK-8, Dojindo Laboratories, Japan) as described previously [6]. MG-63 osteoblast-like cells were seeded on different Ti surfaces or tissue culture plastic (TCP) controls at a density of 2 × 10^4^ cells per well and cultured for 2 and 5 days. Afterward, CCK-8 reagent was added into each well, and plates were incubated at 37 °C for the additional 2 h. Four aliquots of 100 µl were transferred from each well into a new 96-well plate, and the optical density at 450 nm was measured using a microplate reader (Molecular Devices, Sunnyvale, USA).

### Quantitative real-time PCR

The expression levels of different osteogenesis and bone-turnover-related genes in MG-63 osteoblast-like cells were quantified by real-time PCR similarly to methods described previously [30, 31]. MG-63 cells were seeded on titanium disks at a density of 2 × 10^4^ cells in 0.5 ml of MEM and cultured for 2 and 5 days. Isolation of mRNA, transcription into cDNA, and qPCR were performed using the TaqMan_Gene Expression Cells-to-CT^TM^ kit (Ambion/Applied Biosystems, Foster City, CA, USA). qPCR was performed on an ABI StepOnePlus device (Applied Biosystems) in paired reactions using the Taqman gene expression assays with following ID numbers (Applied Biosystems) according to the manufacturer’s instructions: alkaline phosphatase (ALP), Hs01029141_g1; osteocalcin (OC), Hs00609452_g1; OPG, Hs00171068_m1; COL1, Hs00164004_m1; receptor activator of nuclear factor kappa-B ligand (RANKL), Hs00243522_m1; and GAPDH, Hs99999905_m1 that was used as a housekeeping gene. The PCR reactions were done in duplicates and the following thermocycling conditions: 95 °C for 10 min; 40 cycles, each for 15 s at 95 °C, and 1 min at 60 °C. The point at which the PCR product was first detected above a fixed threshold (cycle threshold, Ct), was determined for each sample. Alterations in the expression of target genes were calculated using the 2^−ΔΔCt^ method, where ΔΔCt = (*C*_*t*_^target^ − *C*_*t*_^GAPDH^)_sample_ − (*C*_*t*_^target^ − *C*_*t*_^GAPDH^)_control_, taking MG-63 cells grown on TCP as a control.

### Enzyme-linked immunosorbent assay (ELISA)

Commercially available ELISA kits were used for measurements of OC (BioSource International, Inc., Camarillo, CA, USA) and OPG (Biomedica, GmbH & CoKG, Vienna, Austria) in the supernatants. For the measurement of OC and OPG, the samples were diluted 1:10 and 1:50, respectively. The testing procedure was conducted according to the manufacturer’s instructions using a multi-mode reader Synergy HTX (Biotek, Winooski, VT, USA).

### Scanning electron microscopy

MG-63 cells were seeded on the different surfaces at a density of 2 × 10^4^ cells per well in 0.5 ml of MEM and cultured for 2 days. Afterward, disks were rinsed with PBS, and cells were fixed with 4% formalin for 24 h. Samples were dehydrated with gradient concentrations of ethanol and hexamethyldisilazane (Sigma-Aldrich, St.Louis, MO, USA). Finally, the discs were sputtered with gold for 30 s using the EM ACE200 sputtering device (Leica, Wetzlar, Germany). Samples were analyzed by a scanning electron microscopy (FEI Quanta 200) at an accelerating voltage of 15–20 kV.

### Statistical analysis

Normal data distribution was confirmed by K-S test. The statistical differences between the effects of the various titanium surfaces on the cultured cells were analyzed by one-way analysis of variance for repeated measures followed by *t*-test. All statistical analyses were performed using SPSS (Statistics Software v 19.0; IBM Corp, Armonk, NY). Differences were considered to be statistically significant at *p* < 0.05. Data are expressed as mean ± standard error of mean (SEM). All experiments were repeated at least three times.

## Results

### Scanning electron microscopy

**Fig. 1:**
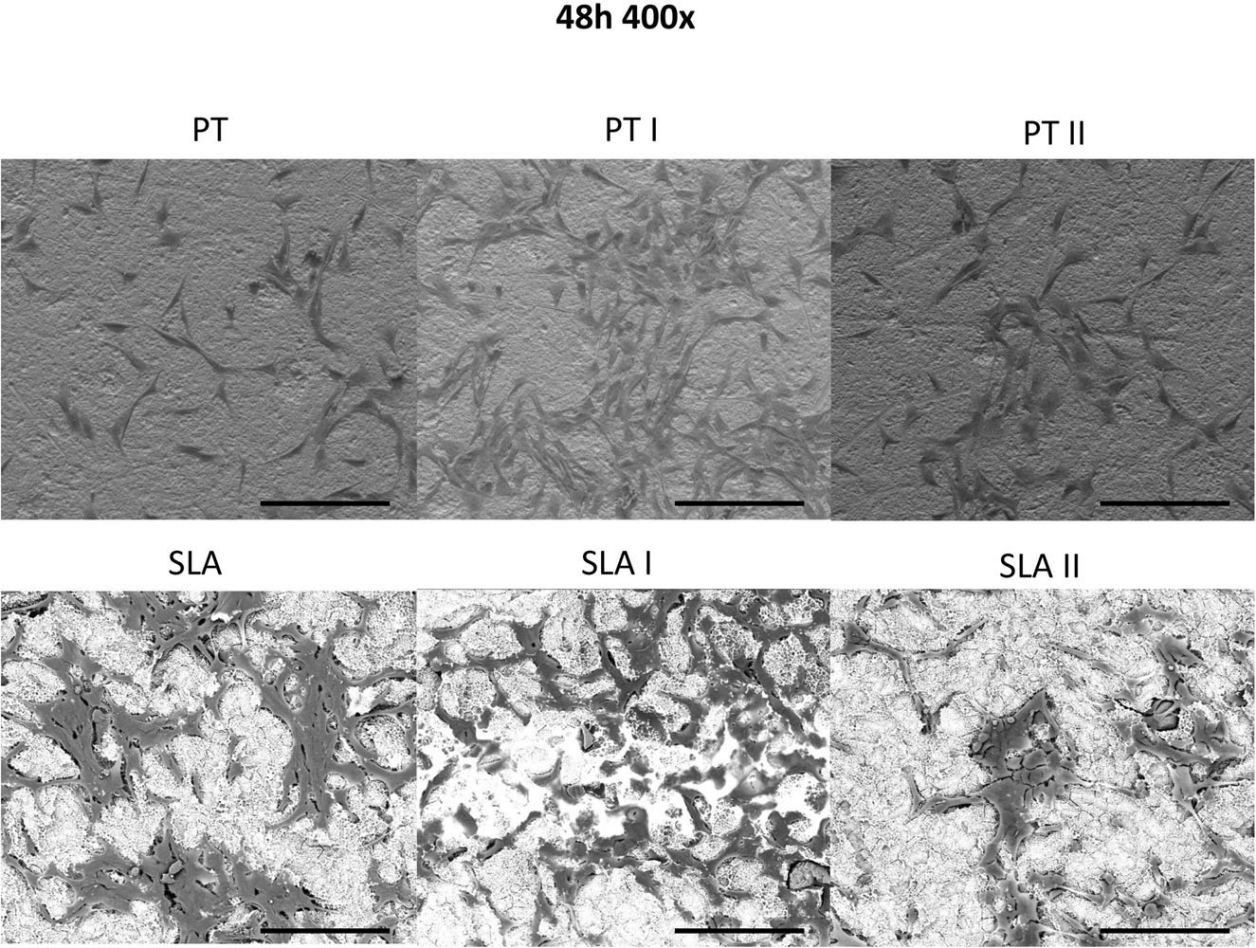
Attachment of osteoblast-like cells on PT surfaces after laser irradiation. MG-63 cells were cultured on smooth or moderately rough surfaces treated with Er:YAG laser with 160 mJ/pulse at 20 Hz (PT I and SLA I), 80 mJ/pulse at 20 Hz (PT II and SLA II) or untreated (PT and SLA) surfaces for 2 days and analyzed by scanning electron microscopy (×400). Scale bars correspond to 200 µm

Scanning electron microscopy pictures of MG-63 cells grown on untreated and Er:YAG laser-irradiated PT and SLA surfaces are presented in Fig. 1. Cells grown on PT surfaces showed no visible changes upon laser irradiation at both settings. Moderately rough surfaces showed macro- and microscopic differences, and melted areas representing the course of the laser spot action were observed at 160 mJ/20 Hz laser treatment (SLA I). At the same settings (SLA I), MG-63 cells appeared condensed in the core area affected by laser irradiation, which can also be observed in SLA II to a lower extent (Fig. 1).

### Proliferation/viability of MG-63 cells on titanium surfaces following laser treatment

**Fig. 2:**
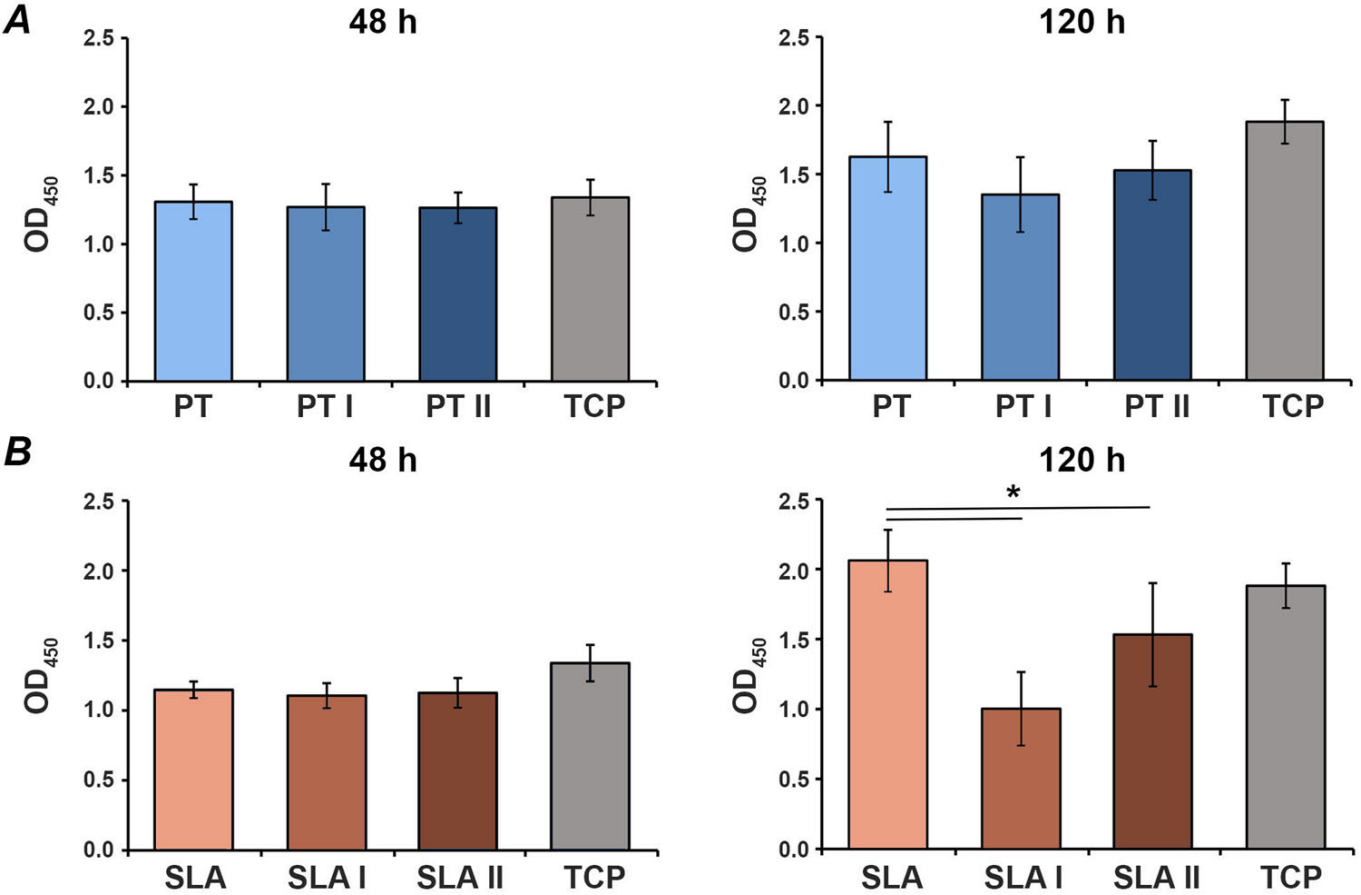
Proliferation/viability of MG-63 cells grown on different titanium surfaces irradiated with Er:YAG laser. MG-63 cells were cultured on smooth (**A**) or moderately rough (**B**) surfaces treated with Er:YAG laser with 160 mJ/pulse at 20 Hz (PT I and SLA I), 80 mJ/pulse at 20 Hz (PT II and SLA II) or untreated surfaces (PT and SLA), and proliferation/viability was measured after 2 and 5 days by CCK-8 method. Cells grown on tissue culture plastic (TCP) served as control. *Y*-axis represents the optical density (OD) values measured at 450 nm. Data are presented as mean ± SEM of three independent experiments

Figure 2 shows the proliferation of MG-63 cells grown on untreated and laser-irradiated surfaces PT and SLA surfaces. There were no significant differences in the proliferation/viability between untreated surfaces and TCP group. Proliferation/viability of MG-63 cells grown on a smooth PT surface was not affected by laser irradiation. In contrast, cells grown on irradiated SLA I and SLA II surfaces exhibited lower proliferation compared to untreated SLA surface after 5 days of culture. No significant difference was observed between the surfaces treated with the different irradiation intensities (Fig. 2).

### Expression of osteogenic differentiation markers in MG-63 osteoblast-like cells

**Fig. 3:**
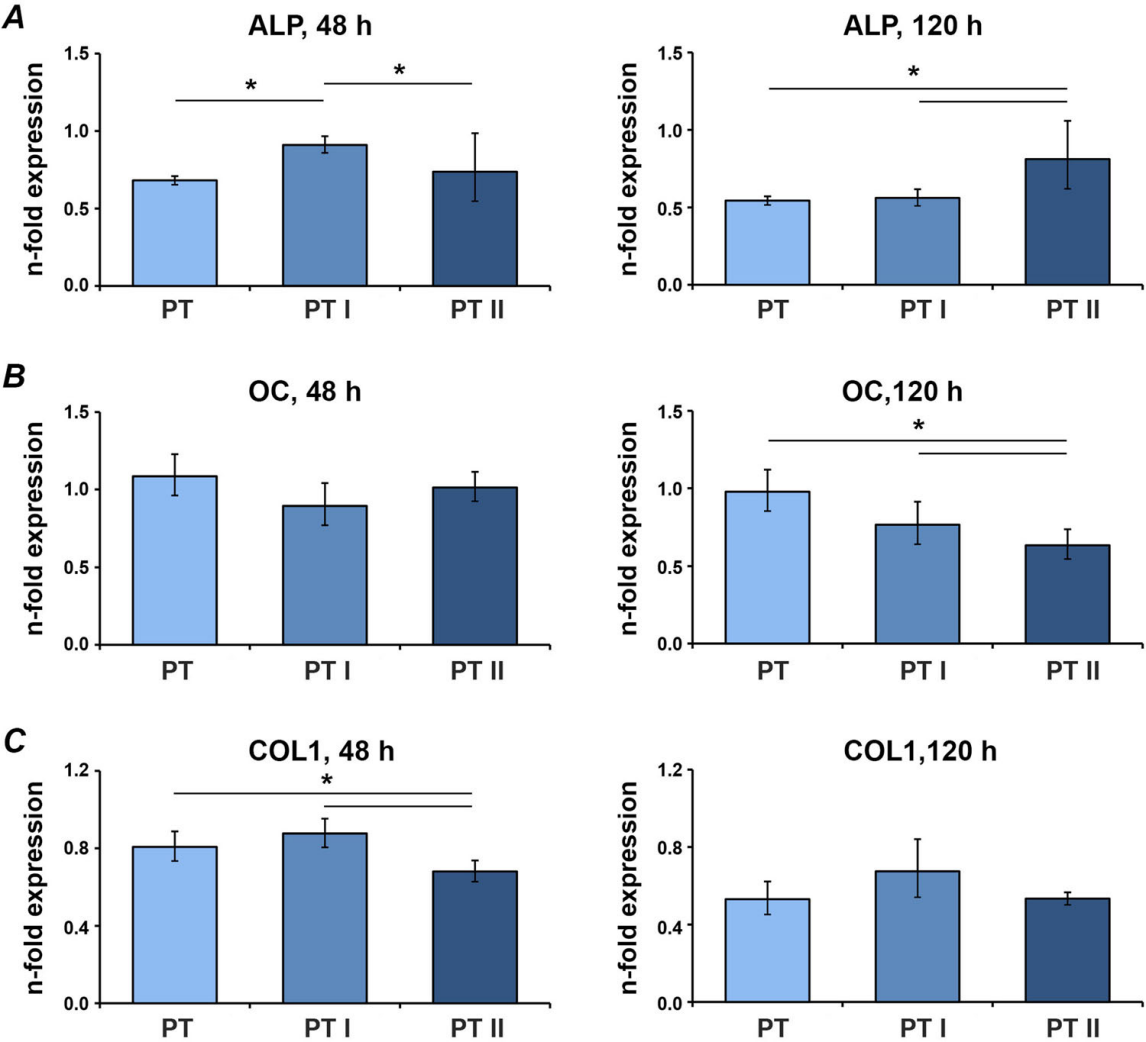
Expression of osteogenesis-related genes in MG-63 grown on smooth surfaces irradiated with Er:YAG laser. MG-63 cells were cultured on PT, PT I, and PT II surfaces for 2 and 5 days, and the expression levels of ALP (**A**), OC (**B**), and COL1 (**C**) were measured by quantitative real-time PCR. *Y*-axis represents *n*-fold expression related to MG-63 grown on tissue culture plastic calculated using 2^−^^ΔΔCt^ method, using GAPDH as housekeeping gene. Data are presented as the mean ± SEM of four independent experiments

**Fig. 4:**
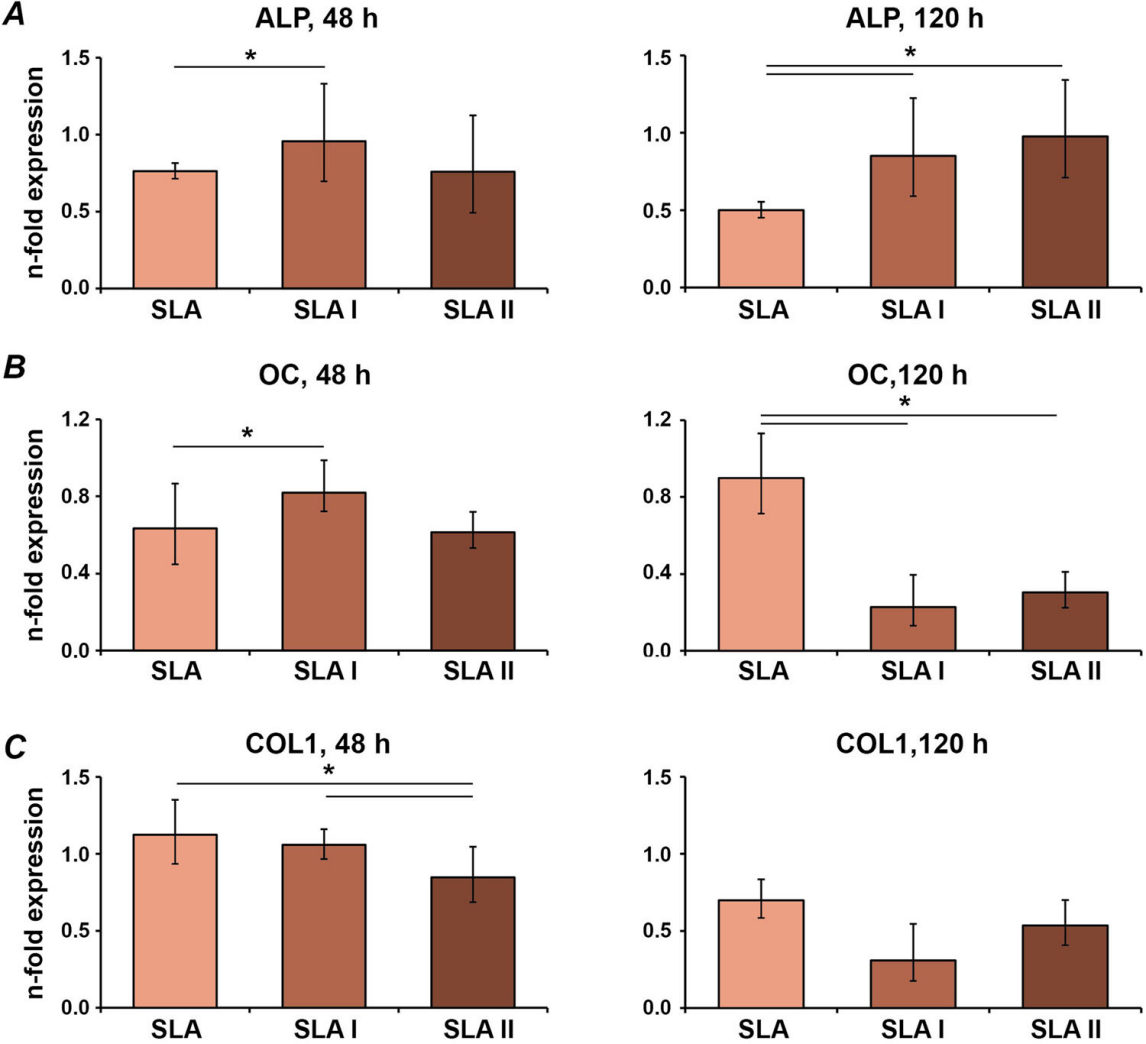
Expression of osteogenesis-related genes in MG-63 grown on moderately rough surfaces irradiated with Er:YAG laser. Following cell culture of MG-63 cells for 2 and 5 days on SLA, SLA I, and SLA II surfaces, the expression levels of ALP (**A**), OC (**B**), and COL1 (**C**) were measured by quantitative real-time PCR. *Y*-axis represents *n*-fold expression related to MG-63 grown on tissue culture plastic calculated using 2^−ΔΔCt^ method, using GAPDH as housekeeping gene. Data are presented as the mean ± SEM of four independent experiments

Gene expression levels of osteogenic factors in MG-63 cells grown on untreated and differently treated PT and SLA surfaces are shown in Figs. 3 and 4, respectively. After 2 days of incubation, gene expression of ALP was significantly higher in cells on PT I compared to PT and PT II surfaces, and COL1 was expressed lower on PT II than on PT, and PT I. After 5 days, ALP gene expression on PT II was significantly higher than on PT and PT I, and OC expression on both laser-irradiated PT surfaces was lower compared to untreated PT surface (Fig. 3). In SLA surfaces after 2 days, gene expression of ALP and OC was increased on SLA I compared to SLA surfaces. OC expression on SLA I was also higher than on SLA II. Furthermore, gene expression of COL1 was lower on SLA II compared to SLA I and SLA surface. After 5 days, gene expression of ALP was higher on both SLA I and SLA II surfaces compared to the untreated SLA surface. Moreover, ALP gene expression on SLA II was higher than on SLA I surfaces. Laser irradiation at both intensities yielded lower OC expression than untreated control. No difference in COL1 expression was observed after 5 days (Fig. 4).

### OC content in conditioned media

**Fig. 5:**
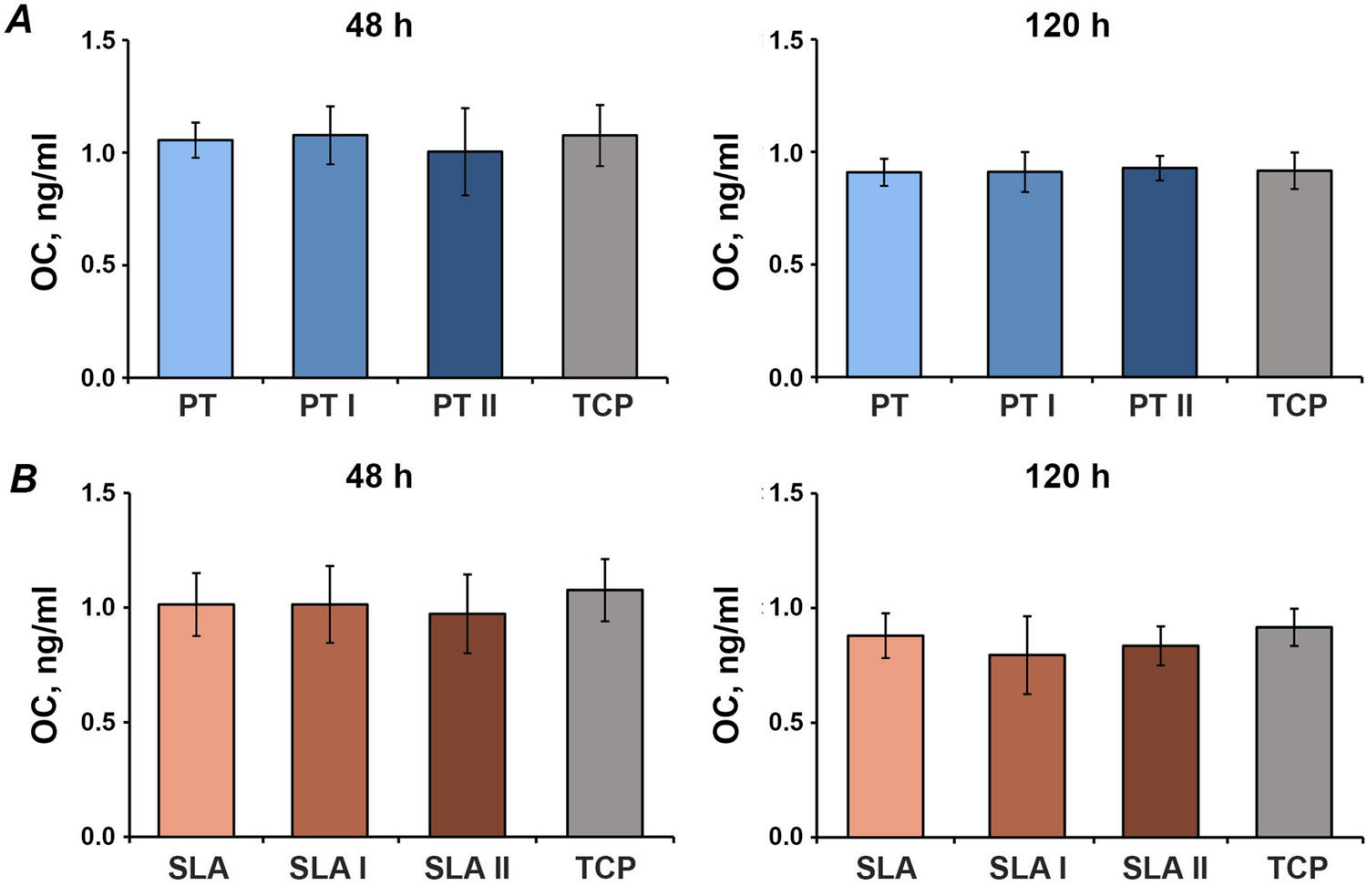
Osteocalcin production by MG-63 cells grown on titanium disks irradiated with Er:YAG laser. MG-63 cells were cultured on smooth (**A**) or moderately rough (**B**) surfaces treated with Er:YAG laser with 160 mJ/pulse at 20 Hz (PT I and SLA I), 80 mJ/pulse at 20 Hz (PT II and SLA II) or untreated surfaces (PT and SLA) for 2 and 5 days, and the levels of OC protein in conditioned media were measured by commercially available ELISA. Data are presented as mean ± SEM of five independent experiments

Figure 5 shows the content of OC protein in the conditioned media of MG-63 cells grown on untreated and laser-irradiated surfaces. No significant differences in the OC content after 2 and 5 days were observed between cells grown on the differently treated surfaces (Fig. 5).

### Expression of bone turnover markers in MG-63 osteoblast-like cells

**Fig. 6:**
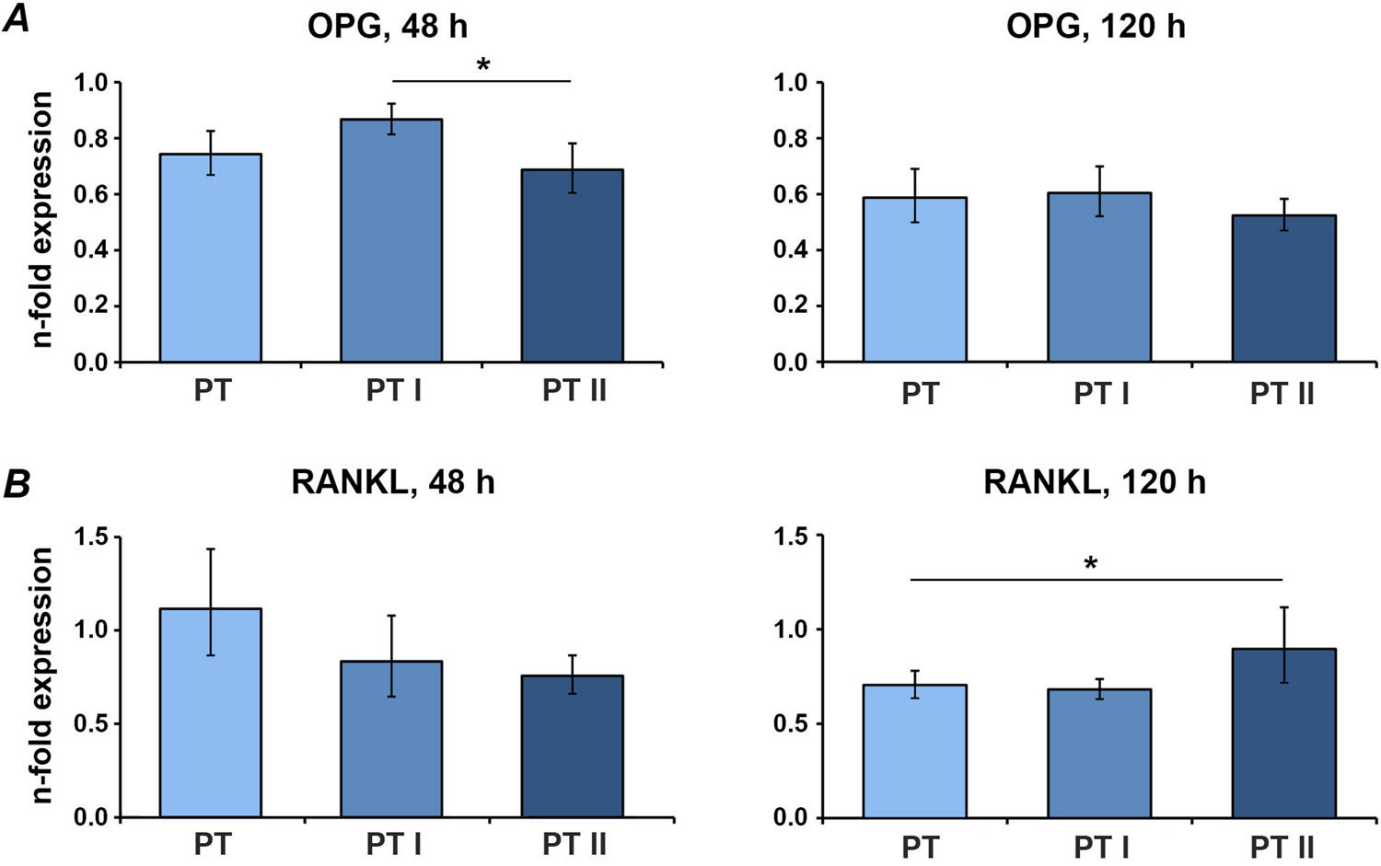
Expression of bone-turnover-related genes in MG-63 grown on smooth surfaces irradiated with Er:YAG laser. MG-63 cells were cultured on smooth surfaces treated with Er:YAG laser with 160 mJ/pulse at 20 Hz (PT II), 80 mJ/pulse at 20 Hz (PT II) or untreated (PT) surfaces for 2 and 5 days, and the expression levels of OPG (**A**) and RANKL (**B**) were measured by quantitative real-time PCR. *Y*-axis represents *n*-fold expression related to MG-63 grown on tissue culture plastic calculated using 2^−ΔΔCt^ method, using GAPDH as housekeeping gene. Data are presented as the mean ± SEM of four independent experiments

**Fig. 7:**
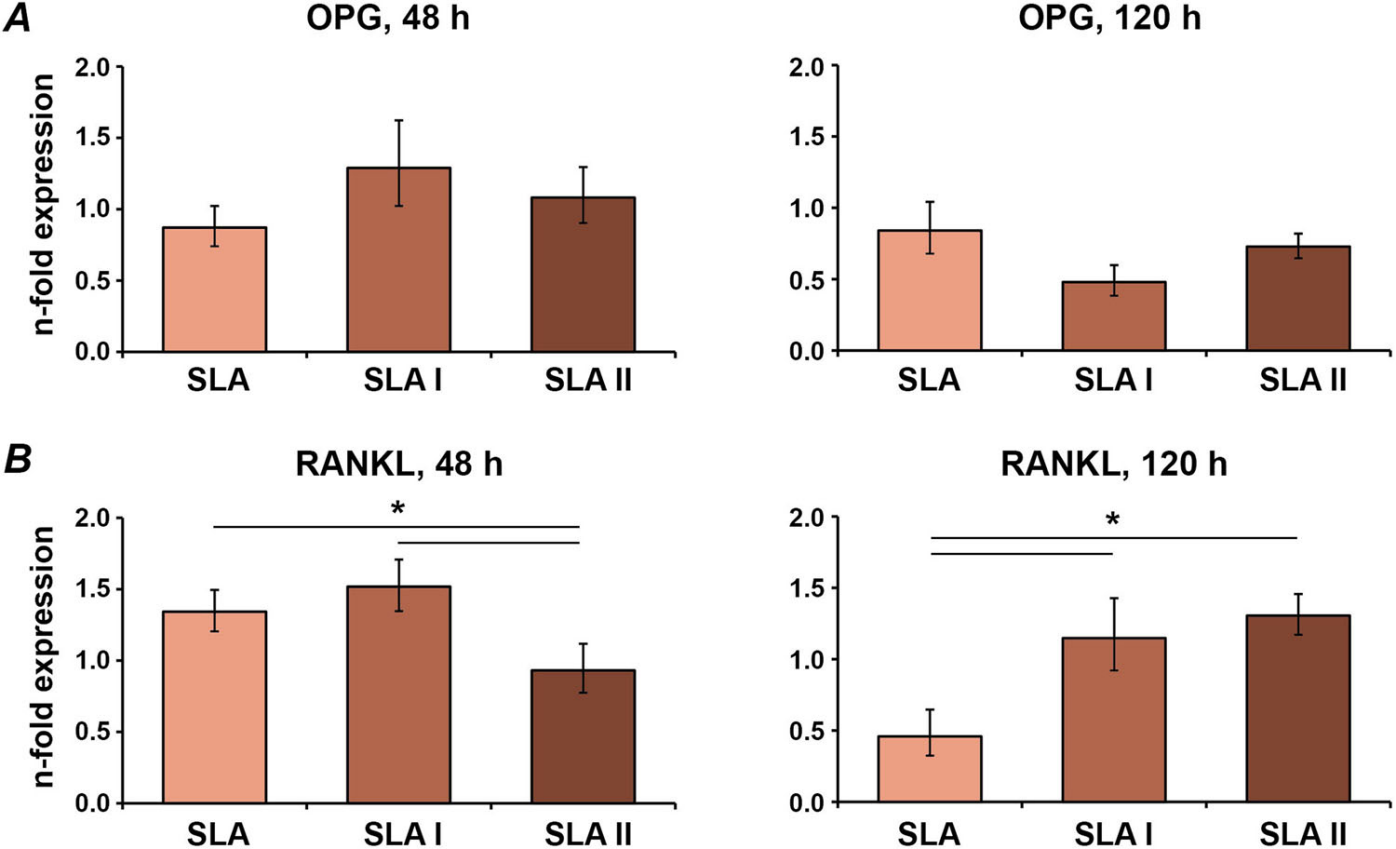
Expression of bone-turnover-related genes in MG-63 grown on moderately rough surfaces irradiated with Er:YAG laser. MG-63 cells were cultured on moderately rough surfaces treated with Er:YAG laser with 160 mJ/pulse at 20 Hz (SLA I), 80 mJ/pulse at 20 Hz (SLA II) or untreated (SLA) surfaces for 2 and 5 days, and the expression levels of OPG (**A**) and RANKL (**B**) were measured by quantitative real-time PCR. *Y*-axis represents *n*-fold expression related to MG-63 grown on tissue culture plastic calculated using 2^−ΔΔCt^ method, using GAPDH as housekeeping gene. Data are presented as the mean ± SEM of four independent experiments

Gene expression of the bone turnover proteins OPG and RANKL in MG-63 cells grown on the untreated and laser-irradiated PT and SLA surfaces is shown in Figs. 6 and 7, respectively. In cells cultivated on smooth surfaces for 2 days, OPG gene expression was higher on PT I compared to PT II, however, both laser-irradiated groups exhibited no difference compared to the untreated control. On PT II, RANKL gene expression was higher than on PT after 5 days (Fig. 6). In cells growing on SLA surfaces for 2 days, RANKL gene expression was significantly lower on SLA II compared to SLA I and untreated SLA. No significant difference in the gene expression of OPG was observed. After 5 days, there was no difference in OPG expression among all surfaces, however, RANKL expression in laser-irradiated groups SLA I and SLA II were higher than non-treated control (Fig. 7).

### OPG production in MG-63 osteoblast-like cells

**Fig. 8:**
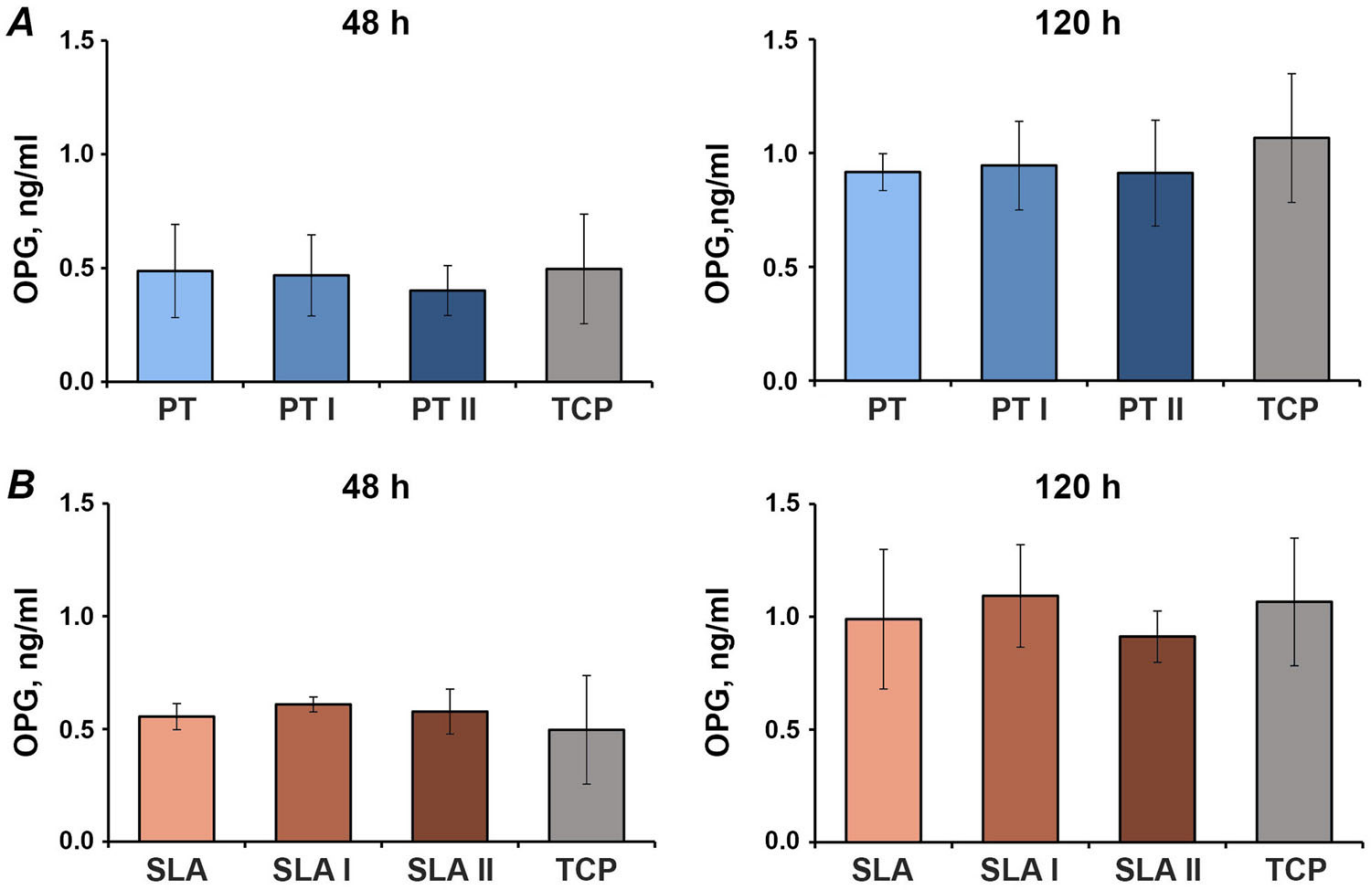
Osteoprotegerin production by MG-63 cells grown on titanium disks irradiated with Er:YAG laser MG-63 cells was cultured on smooth (**A**) or moderately rough (**B**) surfaces treated with Er:YAG laser with 160 mJ/pulse at 20 Hz (PT I and SLA I), 80 mJ/pulse at 20 Hz (PT II and SLA II) or untreated (PT and SLA) surfaces for 2 and 5 days, and the levels of OC protein in conditioned media were measured by commercially available ELISA. Data are presented as mean ± SEM of five independent experiments

Figure 8 shows the concentration of OPG in the conditioned media of MG-63 cells grown on the untreated and laser-irradiated PT and SLA surfaces for 2 and 5 days. OPG protein production was unaffected by laser irradiation (Fig. 8).

## Discussion

Surface decontamination of dental implants is a key requirement in the treatment of biofilm-associated peri-implant diseases such as peri-implant mucositis and peri-implantitis [32]. Although studies have investigated the impact of Er:YAG laser irradiation on titanium surfaces and reported also bactericidal properties [27, 33], evidence on the impact of laser treatment on the topography of Ti surfaces and response of osteogenic cell growing on those surfaces has been rarely shown so far. However, re-osseointegration plays a critical role in the clinical outcome following laser therapy at implant sites affected by peri-implantitis.

The present study demonstrates that irradiation of moderately rough titanium surfaces using the Er.YAG laser impacted the cell morphology of MG-63 osteoblast-like cells that were afterwards cultivated on those surfaces. This effect, however, was not observed for smooth surfaces. The laser parameters used in this study represent actual settings that are applied in patient treatment, and a similar range of settings has been used frequently in clinical trials [34, 35]. Some previous studies already reported the alteration of Ti surface morphology after laser irradiation, notably, Galli et al. [24] using Er:YAG laser with slightly different settings (200 mJ/pulse at 10 Hz) showed that irradiation alters the morphology of sandblasted and acid-etched but not that of machined surfaces. Likewise, Stubinger et al. [23] also reported the alteration of Ti surface morphology by Er:YAG laser used at higher intensities (300 mJ/10 Hz). In contrast, Shin et al. [36] showed that SLA surfaces did not exhibit significant changes of surface texture upon laser irradiation time of fewer than 2 min at power up to 140 mJ/pulse. Interestingly, we observed the irradiation of Er:YAG laser with 80 mJ/pulse and 20 Hz caused changes in the SLA surface topography. Thus, titanium surfaces seem sensitive to changes in irradiation settings such as irradiation time or feed speed, which have to be considered a crucial factor during laser application in clinical treatment.

According to the present findings, laser irradiation of moderately rough Ti surfaces at 160 mJ/20 Hz negatively affects osteoblast proliferation/viability as measured by CCK-8 assay, which is based on the measurements of cell mitochondrial activity and therefore reflects the combination of two functional parameters, cell proliferation, and cell viability. Decreased osteoblasts proliferation on moderately rough Ti surface after laser irradiation might imply its negative effect on the initial osteogenic process. Our results are supported by Galli et al. [24], who also reported that Er:YAG irradiation at somewhat different settings negatively influenced the proliferation/viability of osteoblast-like cells. In contrast, two other studies showed a significantly higher osteoblasts proliferation/viability upon laser irradiation [37, 38]. In this context, it has to be taken into account that all of the referred studies have been performed with Saos-2 cells, an osteosarcoma cell line that might exhibit different traits than the MG-63 osteoblast-like cells used in the present study. MG-63 cells are an osteosarcoma cell line with osteoblastic features and have been demonstrated to keep stable properties during passaging [39, 40], whereas primary osteoblasts may exhibit different traits depending on the source, donor, and isolation method [41].

Laser treatment seems to have partly influenced the expression of osteogenesis-related genes in MG-63 cells, depending on the type of titanium surface. In our study, we focused on the expression of ALP, OC, and collagen type 1. These proteins play an essential role in osteogenesis and bone formation. ALP is considered a marker for early osteogenic differentiation and is detected in high levels in cells that mineralize their matrix, such as osteoblasts [42]. OC is the most abundant non-collagenous protein in bone and is crucial for bone formation and remodeling [43]. Collagen type 1 is a bone matrix protein secreted by osteoblasts and used as a marker for osteoblast differentiation [44]. Although we observed lower gene expression of OC following laser irradiation in moderately rough surfaces, our results suggest that the production of OC on protein level was generally unaffected by laser irradiation in both surface groups at 2 and 5 days compared to untreated control. These findings again are supported by Galli et al. [24], who used similar irradiation settings (150 mJ/10 Hz pulse), investigating OC production in osteoblast-like cells after 3 and 6 days.

Some effects of laser irradiation on the OPG/RANKL ratio in MG-63 cells were observed. After 5 days, RANKL gene expression was higher on laser-irradiated moderately rough surfaces compared to untreated control. OPG and RANKL are critical factors involved in bone metabolism [45]. By activating osteoclasts, RANKL promotes bone resorption, whereas OPG is known to bind to RANKL, thus inhibiting its ability to activate osteoclasts. Therefore, a lower OPG/RANKL ratio might indicate higher bone resorption on the moderately rough surface after laser irradiation. Unfortunately, we could not prove the alteration of the OPG/RANKL ratio on the protein level because the content of RANKL in conditioned media was below the detection limit. The data on OPG protein were in agreement with PCR data, and there was no effect of laser irradiation on OPG production. Although our data imply the alteration of bone turnover upon laser irradiation in the in vitro setting, the clinical relevance of this effect should be confirmed by further studies.

One limitation of the study might be a relatively low number of independent experiments. We repeated cell viability/proliferation experiments three times, which agrees with previous studies using a similar design (e.g., [24, 46] who performed three repetitions). PCR and ELISA experiments were repeated four and five times, respectively, because these experiments showed more considerable variability. Another potential limitation is that we used the osteosarcoma MG-63 cell line. Although these cells largely reflect the properties of osteoblasts [40], our findings should be further confirmed by the experiments with primary osteoblasts or mesenchymal stromal cells.

## Conclusion

In general, the use of Er:YAG laser for surface decontamination aims to perform debridement of implant surfaces from bacteria, without impairing the surface itself. The present findings only partly reflect clinical conditions and might be repeated utilizing biofilm-contaminated titanium surfaces, which was beyond the focus of this research project. Taken together, we conclude that the use of Er:YAG laser for decontamination of titanium surfaces does not impair the expression of specific osteogenesis marker at the gene and protein level; however, it might influence bone turnover. Therefore, the application has to be performed with caution, as inappropriate high laser irradiation might compromise implant re-osseointegration into surrounding bone.
